# ANLN truncation causes a familial fatal acute respiratory distress syndrome in Dalmatian dogs

**DOI:** 10.1371/journal.pgen.1006625

**Published:** 2017-02-21

**Authors:** Saila Holopainen, Marjo K. Hytönen, Pernilla Syrjä, Meharji Arumilli, Anna-Kaisa Järvinen, Minna Rajamäki, Hannes Lohi

**Affiliations:** 1 Department of Veterinary Biosciences, University of Helsinki, Helsinki, Finland; 2 Research Programs Unit, Molecular Neurology, University of Helsinki, Helsinki, Finland; 3 The Folkhälsan Institute of Genetics, Helsinki, Finland; 4 Department of Equine and Small Animal Medicine, University of Helsinki, Helsinki, Finland; Stanford University School of Medicine, UNITED STATES

## Abstract

Acute respiratory distress syndrome (ARDS) is the leading cause of death in critical care medicine. The syndrome is typified by an exaggerated inflammatory response within the lungs. ARDS has been reported in many species, including dogs. We have previously reported a fatal familial juvenile respiratory disease accompanied by occasional unilateral renal aplasia and hydrocephalus, in Dalmatian dogs. The condition with a suggested recessive mode of inheritance resembles acute exacerbation of usual interstitial pneumonia in man. We combined SNP-based homozygosity mapping of two ARDS-affected Dalmatian dogs and whole genome sequencing of one affected dog to identify a case-specific homozygous nonsense variant, c.31C>T; p.R11* in the *ANLN* gene. Subsequent analysis of the variant in a total cohort of 188 Dalmatians, including seven cases, indicated complete segregation of the variant with the disease and confirmed an autosomal recessive mode of inheritance. Low carrier frequency of 1.7% was observed in a population cohort. The early nonsense variant results in a nearly complete truncation of the ANLN protein and immunohistochemical analysis of the affected lung tissue demonstrated the lack of the membranous and cytoplasmic staining of ANLN protein in the metaplastic bronchial epithelium. The *ANLN* gene encodes an anillin actin binding protein with a suggested regulatory role in the integrity of intercellular junctions. Our study suggests that defective ANLN results in abnormal cellular organization of the bronchiolar epithelium, which in turn predisposes to acute respiratory distress. *ANLN* has been previously linked to a dominant focal segmental glomerulosclerosis in human without pulmonary defects. However, the lack of similar renal manifestations in the affected Dalmatians suggest a novel ANLN-related pulmonary function and disease association.

## Author summary

Acute respiratory distress syndrome (ARDS) is characterized by life-threatening impairment of pulmonary gas exchange and leads to substantial mortality in man. Spontaneous ARDS has also been described in dogs including a familial fatal ARDS-like syndrome in young Dalmatian dogs. The main clinical signs include progressive tachypnea and dyspnea leading to a severe respiratory distress and euthanasia. The prominent clinicopathological findings involve pulmonary lesions, although some affected puppies also presented with renal aplasia and hydrocephalus. This study finds the genetic cause of the disease by identifying a recessive nonsense variant in the *ANLN* gene. The *ANLN* gene encodes an anillin actin binding protein which has an important role in the integrity of the epithelial cell organization. The functional defect of ANLN due to early truncation and absence from the bronchiolar epithelium is consistent with the observed histopathology with hyper- and metaplasia of the bronchiolar epithelium and clinical ARDS. Our study reveals a novel pulmonary disease association for *ANLN*, provides new insights to pathophysiology and has enabled the development of a genetic test for breeding purposes.

## Introduction

Acute respiratory distress syndrome (ARDS) is a multifactorial syndrome characterized by rapid-onset respiratory failure resulting from pulmonary inflammation [1]. ARDS is common and leads to substantial mortality in man [1]. Two forms of idiopathic interstitial pneumonia are found in human ARDS: a diffuse alveolar damage (DAD) in acute interstitial pneumonia (AIP) and an acute exacerbation of usual interstitial pneumonia in idiopathic pulmonary fibrosis [2].

The molecular mechanisms leading to human ARDS remain largely unknown. Candidate gene studies suggest the involvement of inflammatory mediators such as interleukins IL-6, IL-8 and IL-32 [3,4], pre-B-cell colony-enhancing factor (PBEF) [5,6], and angiotensin-converting enzyme (ACE) [7]. In addition, the nuclear factor erythroid-derived 2–like 2 (NFE2L2) transcription factor has been identified as a potential mediator of acute lung injury in a mouse model [8].

Spontaneous ARDS has also been described in dogs [9]. We previously described a familial fatal ARDS-like syndrome in young Dalmatian dogs with the main clinical signs including progressive tachypnea and dyspnea leading to severe respiratory distress and euthanasia [10]. The clinicopathological findings were restricted to pulmonary lesions in the majority of the affected Dalmatians, although some of the puppies presented with concurrent unilateral renal aplasia and hydrocephalus [10]. Pulmonary manifestations included multiple foci of marked atypical hyperplasia and squamous metaplasia of the bronchiolar epithelium, patchy ongoing fibrosis with myofibroblastic metaplasia, smooth muscle hyperplasia and occasional honeycombing of alveoli and hyperplasia of type II pneumocytes (PCII) along with acute alveolar edema [11].

Exclusion of specific causes like exposure to toxins and viruses and overrepresentation of the cases in the Dalmatian breed suggested a recessive genetic defect [10], which we aimed to reveal in this study. We describe the identification of a fully penetrant recessive nonsense variant in a novel candidate gene, *ANLN*. The *ANLN* gene encodes an anillin actin binding protein which has an important role in the integrity of the epithelial cell organization. The functional defect of ANLN due to early truncation is consistent with the observed histopathology with hyper- and metaplasia of the bronchiolar epithelium, consecutive DAD and clinical ARDS.

## Results

### Identification of a nonsense variant in the *ANLN* gene

**Fig 1:**
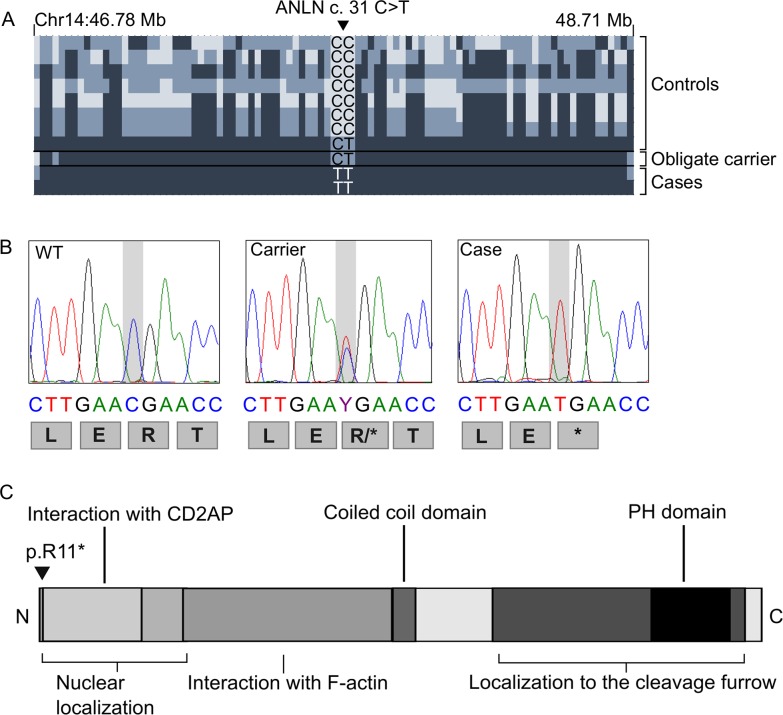
Identification of a nonsense variant in the *ANLN* gene. (A) The *ANLN* c.31C>T nonsense variant found within a 2 Mb homozygosity region on chromosome 14. Haplotypes are shown for eleven Dalmatians (two cases, an obligate carrier and eight controls) genotyped with canine HD SNP array. Colors in haplotype block are as follows: dark blue marks the homozygous allele, blue heterozygous allele and light blue an opposite homozygous allele. (B) Chromatograms showing the genotypes and predicted translations of *ANLN* c.31C>T variant for a wild-type, a carrier and an affected dog after Sanger sequencing. (C) A schematic presentation of the ANLN protein structure. The identified early truncation mutation p.R11* is indicated by arrow.

To identify the genetic cause of ARDS in Dalmatians, we performed a combined analysis of homozygosity mapping and whole genome sequencing (WGS). The study cohort of eleven Dalmatians including two affected littermates, one healthy obligate carrier, one healthy sibling, one healthy grandparent and six other healthy dogs were genotyped using Illumina’s CanineHD SNP array. Genotype data of two cases was used for homozygosity mapping, which revealed 49 shared homozygous regions (S1 Table). Whole genome sequencing with mean coverage of 16x was performed on one affected dog. The filtering of variants from WGS data under recessive model of inheritance against WGS and exome variant data of 136 unaffected dogs (S2 Table) uncovered 16,195 case-specific variants of which 98 were exonic (Table 1). Only 15 out of the 98 coding variants were found in the homozygosity regions of which eight variants were either non-synonymous (n = 4), frameshift (n = 3) or nonsense (n = 1) (Table 2). Manual inspection of the two *PSMD6* deletions with Integrated Genome Viewer (IGV) revealed that the variants were also present in several control genomes (S2 Table), excluding the gene as a candidate for the disease. The other six exonic variants in *ANLN*, *CD302*, *GANAB*, *MUC5B*, *OR16D05* and *ORO8C02* genes (Table 2) were genotyped in a cohort of twelve dogs, including seven cases and five closely related unaffected dogs (parent, grandparent and three healthy siblings). Variants in the *CD302*, *GANAB*, *MUC5B*, *OR16D05* and *ORO8C02* genes were excluded as they did not agree with a recessive segregation pattern. A complete segregation was found only for the c.31C>T variant in the *ANLN* gene. All seven affected dogs were homozygous, both the parent and the grandparent were heterozygous, and healthy littermates were either heterozygous (1/3) or wild-type (2/3) (Fig 1A and 1B).

**Table 1:** Summary of the variants after various filtering and validation steps using the WGS data from an affected Dalmatian dog.

Variant filtering steps	Number of variants
Total number of variants in the Dalmatian WGS	6,280,192
Variants that segregate under recessive mode of inheritance	16,195
Variants in the exonic regions (Ensembl annotation)	98
Variants in the 49 identified regions of homozygosity	15
Non-synonymous, frameshift and nonsense variants in the regions of homozygosity	8
Experimentally validated case-specific coding variants (7 cases and five controls)	1

**Table 2:** Summary of eight case-specific coding variants in the identified 49 regions of homozygosity.

Chr	Position (bp)	Ref. allele	Alt. allele	Gene	Variant (transcript)	Variant (protein)	Transcript reference	Effect
14	47,812,143	C	T	*ANLN*	exon 2: c.31C>T	p.R11*	ENSCAFT00000005209	nonsense
18	54,012,308	G	A	*GANAB*	exon 13: c.1501G>A	p.V501M	ENSCAFT00000025009	missense
18	45,539,139	C	T	*MUC5B*	exon 33: c.13216C>T	p.H4406Y	ENSCAFT00000045408	missense
18	41,555,095	C	T	*OR08C02*	exon 1: c.28G>A	p.V10I	ENSCAFT00000044785	missense
18	40,066,054	TGGGGG	TGGGG	*OR16D05*	exon 1:c.233delC	p.A78fs	ENSCAFT00000012940	frameshift, deletion
20	27,206,732	TGCCGC	T	*PSMD6*	exon 1: c.292_296del	p.A98fs	ENSCAFT00000011009	frameshift, deletion
20	27,206,738	TG	T	*PSMD6*	exon 1: c.292_296del	p.A100fs	ENSCAFT00000011009	frameshift, deletion
36	5,562,515	C	T	*CD302*	exon 1: c.298delG	p.A130T	ENSCAFT00000049520	missense

The *ANLN* c.31C>T variant is located within a 2 Mb region of continuous homozygosity on chromosome 14 at 46.78–48.71 Mb (Fig 1A). The 2 Mb haplotype is part of the larger 14.6 Mb homozygosity region identified by homozygosity mapping (S1 Table). The entire 14.6 Mb homozygosity region had an average 15x coverage (>90% bases with at least 10x coverage) and did not contain other case-specific coding homozygous variants. We manually assessed the haplotypes surrounding the *ANLN* variant in eleven dogs that were genotyped with the SNP array (Fig 1A). The 2 Mb haplotype was homozygous in both cases and in the parent and the grandparent. However, only the two cases were homozygous for the *ANLN* c.31C>T variant while the parent and the grandparent were heterozygous, suggesting a recent origin of the risk variant in the pedigree.

Further validation of the *ANLN* c.31C>T variant in 176 randomly selected unaffected Dalmatian dogs revealed a low 1.7% carrier frequency (3/176). No new genetically affected dogs were found while three new non-Finnish carriers were identified. The pedigree information of these three dogs was not available and therefore the relationship to the original Finnish discovery population remains unknown. A combined analysis in the entire cohorts of 188 dogs (12 +176 Dalmatians) gave a highly significant association between the T allele and the disease (p = 3.075x10^-58^).

Breed-specificity was studied by screening the *ANLN* variant in 31 Pointers, which is considered as the closest relative to Dalmatians. None of the Pointers carried the variant.

The identified *ANLN* c.31C>T nonsense variant is predicted to result in an early truncation of the normal 1121 amino acid ANLN protein after the first ten residues (Fig 1C). This severe truncation very likely completely abrogates the ANLN function.

### Immunohistochemistry indicates lack of cytoplasmic ANLN in the bronchiolar epithelium and pneumocyte type II cells

The effect of the truncation could not be assessed at transcriptional level, as fresh RNA samples from the affected dogs were not available. Therefore, the ANLN expression was analyzed by immunohistochemistry, using antibody recognizing residues 1074–1124 of the protein, expected to be lacking in the affected dogs. Various organs from an age- and breed-matched control dog were included, and the staining was compared to that in the lungs and other tissues available from four affected Dalmatians. In addition, the expression pattern in the lungs of the affected dogs was compared to a canine lung affected by diffuse alveolar damage (DAD) to include the assessment of PCII cells, which are important for normal regeneration process in the alveolus [9]. The kidneys from seven affected Dalmatians were histologically re-evaluated, since loss of ANLN function has been linked to human focal segmental glomerulosclerosis (FSGS) [12]. Glomerular collagen was highlighted by Masson-trichrome staining and basal membranes by periodic acid-Schiff staining (PAS) in order to reveal even subtle fibrosis.

**Fig 2:**
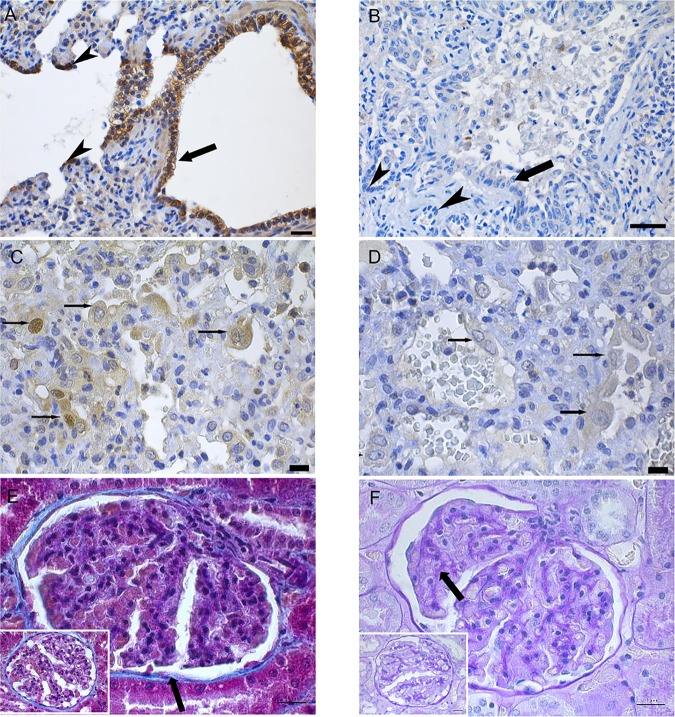
Histological findings of lung and kidneys. (A) In Anillin immunohistochemistry (IHC) at the level of bronchioalveolar junction a strong positive membranous signal is detected in the bronchiolar epithelial cells (arrow) and cytoplasmic labelling is seen in cells lining the respiratory bronchiolus (arrowhead) in a control dog. Scale bar 50 μm. (B) IHC Anillin, affected dog: No membranous signal is detected in the dysplastic bronchiolar epithelium (arrow) nor in the cells lining adjacent alveoli (arrow head) in an affected dog. Scale bar 100 μm. (C) IHC Anillin control dog: PCII cells lining the alveolar septae during re-epithelization show cytoplasmic positivity. Scale bar 20 μm. (D) IHC Anillin, affected dog: PC II cells lack the cytoplasmic positivity. Scale bar 20 μm. (E) The collagen, staining blue in Masson’s Trichome Stain (arrow), of the glomerular tuft is uniform and minimal throughout the glomerulus, similar to that of the control dog (inset). Scale bars 20 μm. (F) Mesangial basal membranes (arrow) staining pink, are slender both in the glomerula of affected and control dog (inset). PAS, scale bars 20 μm.

We found a specific positive membranous ANLN-signal in the epithelial cells lining the terminal bronchioles in the control sample (Fig 2A) and in the control lung affected by DAD. In addition, proliferating PCII cells showed a strong cytoplasmic positivity in the canine lung affected by DAD (Fig 2C). In contrast, the membranous bronchiolar epithelial ANLN-signal as well as the cytoplasmic PCII staining were absent in the lung specimens of the affected Dalmatians (Fig 2B and 2D). Few basal bronchial and alveolar interstitial cells showed nuclear positivity in all groups. In other organs, ANLN expression was neither detected in the control dogs, nor in the tested affected dogs. Glomerular tufts appeared histologically normal, with no excessive collagen deposition (Fig 2E) and slender glomerular basal membranes (Fig 2F). These results support the conclusion that ANLN is mainly expressed in the canine lung, co-localizes with the histological lesion and is lacking in the affected Dalmatians. Specific ANLN expression was not detected in organs other than lung in the canine control samples. Lesions similar to those described in human FSGS were not present in the kidneys of the affected dogs.

## Discussion

This study reveals the primary cause of ARDS in Dalmatian dogs by identifying a recessive nonsense variant (c.31C>T, p.R11*) in the *ANLN* gene. Several findings support the causality of the ANLN defect in ARDS. First, the nonsense variant was the only case-specific coding change within the homozygosity regions that fully segregated with the disease (p = 3.075x10^-58^). Second, the genetic defect results in the very early truncation abolishing the function of the protein. Third, the ANLN protein was found to be expressed predominantly in the lungs, which is the key affected organ in ARDS. ANLN was absent in the lungs of the affected dogs. Finally, *ANLN* is a relevant functional candidate gene, since it plays a role in cell division and in the assembly of intercellular junctions [12–14]. Histopathology of the affected dogs show a disorganized bronchiolar epithelial regeneration attempt and disturbed alveolar epithelial regeneration [10, 11], which could be due to improper ANLN contributions. Therefore, our study has important implications since it uncovers a novel candidate gene for ARDS and sheds new light on the understanding of the underlying pathophysiology.

The *ANLN* gene encodes a 124 kDa intracellular multi-domain protein (Fig 1C) that is expressed in several organs, including lungs, kidney and brain [14–16]. ANLN interacts with F-actin and CD2-associated protein (CD2AP) [12] and is implicated in cytoskeletal dynamics [14]. As ANLN interacts both with the organizing mitotic spindle during mitosis and the cytoskeletal actin in cellular migration [15], the absence of the protein and consequent disturbed migration and proliferation of PCII at the alveolar level could trigger the immense bronchiolar epithelial regeneration attempt seen on the histopathology of the affected Dalmatians. One of the key events in the repair of alveolar injury involves the proliferation and migration of ANLN-positive PCII cells [9]. In addition, the atypical, broad based and multinucleated PCII of the affected dogs correlate morphologically with the disturbed cell division, likely caused by the absence of functional ANLN.

An alternative or complementary hypothesis is that the loss of ANLN results primarily in the disorganized bronchiolar epithelia in the affected Dalmatians due to improper assembly of intercellular junctions. Hereby the hyper-and dysplastic epithelium acts as a mechanical hurdle at the bronchioalveolar junction during expiration and air is trapped at the alveolar level during passive exhalation, causing over-extension damage to the alveolar wall and ARDS. A comparable pathogenesis leads to ARDS in ventilator-induced lung injury in man and dog, where alveolar over-inflation, with consecutive alveolar edema and alveolar emphysema, progresses into interlobular emphysema and pneumomediastinum [9]. Seven of the affected dogs suffered from marked alveolar edema, five from marked alveolar emphysema [10, 11]. In addition, three dogs developed pneumomediastium [10], which is rare in dogs not suffering from perforating trauma of the esophagus, neck or trachea.

*ANLN* has been associated in diverse forms of neoplastic disease in man [15, 17–20] with a proposed role in regulating intercellular adhesion in the epithelia [21]. Interestingly, another scaffold protein Alix has recently been associated with the maintenance of epithelial cell polarity and assembly of intercellular junctions [16]. Abnormal structure of the choroid plexus epithelium and ependymal in the *Alix* knockout mouse results in enlargement of the lateral ventricles and hydrocephalus as the homeostasis of the blood-cerebrospinal fluid barrier requires intact tight junctions. Alix, just like anillin, interacts with F-actin and in addition, with tight junction protein ZO-1, being essential for the maintenance of epithelial cell polarity and barrier. Some of the ARDS-affected Dalmatians manifested also hydrocephalus and renal aplasia [10], which could be caused by abnormal assembly of intercellular junctions in the epithelium of the choroid plexus and the ureteric bud epithelium during early organogenesis.

Mutations in the *ANLN* gene have been linked to human FSGS and ANLN has been suggested to play a role in retaining the podocyte function in the glomerular filtration barrier [12]. We did not identify ANLN in the normal canine glomeruli by IHC staining. These findings are similar to the IHC findings in man, where ANLN expression was upregulated in the glomeruli affected by FSGS but not detected in normal glomeruli. Apart from unilateral renal aplasia in two affected dogs, serum biochemistry and renal histology of the affected Dalmatians did not reveal other renal disease [10, 11]. The suggested conclusion [12] that ANLN expression is induced in response to podocyte injury and repair, not in the end-differentiated mature podocyte, remains unverified in dogs as early onset and lethal outcome of the disease prevents further study of a potential renal phenotype. Unilateral renal aplasia may result from a developmental expression of *ANLN*.

Additional evidence for the physiological significance of the loss-of-function variants in genes can be explored utilizing available variant databases. We searched possible loss-of-function variants (frameshifts and nonsense variants) for canine and human *ANLN* in our canine variant database and in public Genome Aggregation Database (gnomAD) [22]. No loss-of-function variants were found in 136 unaffected dogs while the exploration of the GnomAD database revealed four heterozygous frameshifts and fourteen heterozygous nonsense variants with very low allele frequencies and most being singletons. It therefore appears that the loss-of-function variants in *ANLN* are extremely rare across species, which supports the vital role of the gene for survival and is in agreement with the observed lethal disease in the affected Dalmatians.

In summary, our study reveals a novel lethal pulmonary disease association with the *ANLN* gene and suggests that abnormal cytoskeletal dynamics and epithelial regeneration due to lack of functional ANLN result in the hyper- and metaplastic bronchiolar epithelium that predisposes the affected dogs to ARDS. A genetic test can be established to facilitate veterinary diagnostics and to eradicate the detrimental condition in the affected breed.

## Materials and methods

### Ethics statement

The experiments performed on dogs were approved by the “Animal Ethics Committee at the State Provincial Office of Southern Finland” (permits: ESAVI/6054/04.10.03/2012 and ESAVI/7482/04.10.07/2015, expire date 17.10.2020) and by the “University of Helsinki Viikki Campus Research Ethics Committee” (Statement 4/2014).

### Study cohort

Altogether 188 Dalmatian dogs and 31 Pointers were included in the study. Samples were obtained from seven affected Dalmatian dogs from four litters presented to the Small Animal Hospital of Helsinki University as described previously [10]. The mean age at the onset of illness in the puppies included in this study was seven months (range 5–10 months). The mean duration of illness varied from one to six weeks, with a mean of three weeks. Four of the puppies were male, three female. DNA was extracted either from formalin fixed paraffin embedded (FFPE) tissue samples (four dogs), from bronchoalveolar lavage samples (two dogs) or from whole EDTA -blood of unaffected Dalmatian dogs in Finland. All dogs in this study were privately owned pets that were examined with the owners’ consent.

Genomic DNA from the FFPE and EDTA samples was extracted using the semi-automated Chemagen extraction robot (PerkinElmer Chemagen Technologie GmbH, Germany). DNA from the BAL samples were extracted using QIAamp DNA Micro Kit (Qiagen, Germany). DNA concentration was determined either with the NanoDrop ND-1000 UV/Vis Spectrophotometer (Thermo Fisher Scientific Inc., USA) or Qubit 3.0 Fluorometer (Thermo Fisher Scientific Inc., USA).

### Homozygosity mapping

Genome-wide SNP genotyping of two affected, one healthy sibling, one obligate (parent) and one possible carrier (grandparent) and six healthy control Dalmatian dogs was performed at the GeneSeek facility (Neogen Corporation, USA) using Illumina’s CanineHD BedChips containing 173,662 validated SNPs. Genotypes were stored in BC/Gene database version 3.5 (BC/Platforms). The PLINK v 1.07 software was used to search for segments of extended homozygosity in the two affected dogs as described previously [23, 24]. Genotype data was filtered using a SNP call rate of > 95%, an array call rate > 95% and minor allele frequency of > 0.05. The genotype data is available for further use upon request.

### Whole genome sequencing

We performed WGS of one affected Dalmatian dog and used 136 other dog genomes (48 whole exome sequences and 88 whole genome sequences) available as controls (S2 Table). A fragment library was prepared with a 290 bp insert size and collected to a single lane of Illumina HiSeq2000 paired-end reads (2 x 100 bp). The reads were processed using speedseq align module available in SpeedSeq suite to produce a duplicate-marked, sorted and indexed BAM file. The Genome Analysis Tool kit (version = 3.3.0-g37228af) was used to perform realignment around potential indel sites and base quality score recalibration using the known SNP variation available at the Broad Institute (https://www.broadinstitute.org/ftp/pub/vgb/dog/trackHub/canFam3/variation). Dual algorithms, Samtools mpileup (version samtools-1.2) and GATK haplotype caller module were used to detect variants and the variants from both algorithms were merged into variant call format (VCFv4.1). In summary, 98.37% of the reads from Dalmatian dog were mapped to the reference genome yielding a genome-wide mean coverage of 16X. We identified 1,475,318 indels and 4,804,627 SNPs of which 39.91% of the variants were known and 60.09% were novel compared to SNPs from Axelsson *et al*., Lindblad-Toh *et al*. and Vaysse *et al*. and dbSNP build 131 [25–27]. Annovar and SnpEff tools were used to annotate the variants to Ensembl, NCBI and Broad annotation databases to predict the functional effects of the variants. Canine genome build CanFam 3.1 was used as a reference sequence.

### Sanger sequencing

We used PCR and Sanger sequencing to perform targeted genotyping for selected variants in the candidate gene. PCR primers were designed with Primer 3 [28] to assess the prevalence of the mutation by Sanger sequencing in a cohort of Dalmatian dogs. We performed a standard PCR, including 0.5 U Biotools DNA Polymerase (Biotools, Madrid, Spain), 2.0 mM MgCl_2_ (Biotools, Madrid, Spain), 200 μM dNTPs (Finnzymes, Espoo, Finland), 1 x Biotools PCR Buffer (Biotools, Madrid, Spain), 0.5 μM forward primers and 0.5 μM reverse primers (S3 Table). All primers were custom ordered from Sigma Aldrich (St. Louis, MO, USA). Reaction mixtures were subjected to the thermal cycling program of 95°C for 10 min, 35 cycles of 95°C for 30 s, 30 s 57°C, 72°C for 40 s and final elongation state of 72°C for 10 min. Genomic PCR products were sequenced using a 3730xl DNA Analyzer (Applied Biosystems, Foster City California, USA) in the core facility, Institute for Molecular Medicine Finland (FIMM, Technology Centre, University of Helsinki, Helsinki, Finland). We analyzed the sequence data with Sequencher 5.3 software (Gene Codes Corp, Ann Arbor, MI, USA).

### Bioinformatic analysis

The UniProt database (http://www.uniprot.org) and SMART tool (http://smart.embl-heidelberg.de) were used to confirm the protein domain structure of ANLN [29–30]. The *ANLN* sequence alignment and cross-species conservation was analyzed with ClustalW2 algorithm (http://www.ebi.ac.uk/Tools/clustalw2/). All numbering with the *ANLN* gene correspond to the accessions ENSCAFG00000003243 (gene) and ENSCAFT00000005209 (protein).

### Immunohistochemical staining of ANLN and renal histopathology

Archived paraffin blocks of autopsy tissue samples from four affected Dalmatian dogs were available for immunohistochemical staining. Autopsy lung samples from a 5-month-old, male Dalmatian, euthanized due to epilepsy and without histopathological changes in the lungs and other internal organs was used as healthy controls. Organs available for assessment of the ANLN expression in the normal dog included lung, kidney, smooth and cross-striated muscle, heart, choroid plexus, testis, liver, spleen, pancreas and lymph node. Lung samples from a 5-year-old Chihuahua male, euthanized due to ARDS with histopathologically confirmed AIP and DAD, were used as comparison of PCII expression pattern in wild-type dogs. Paraffin blocks were sectioned at 4 μm thickness and deparaffinized, antigens were retrieved with 0.01M citrate buffer at pH 6 and heat for 20 minutes at 99°C. Overnight- incubation was used for the primary antibody (rabbit-polyclonal anti-Anillin Antibody aa1074-1124, LS-C288200 (LifeSpan BioSciences, Inc., USA). The sections were stained according to the UltraVision Detection System HRP/DAB kit (Thermo Fisher Scientific Inc., USA). Separate tissue sections from all of the dogs were also stained with hematoxylin and eosin (HE). The hematoxylin-eosin stained histological slides of kidneys from seven affected Dalmatians, including two adults, were histologically re-evaluated for a renal phenotype. Paraffin blocks from four puppies were available for further studies of the kidney and glomerular collagen was highlighted by Masson-trichrome (MTC) staining and basal membranes by periodic acid-Schiff (PAS) staining in order to reveal even subtle fibrosis.

## Supporting information

S1 Table Homozygous genome regions with shared alleles among the 2 analyzed cases (raw PLINK output). The positions refer to the CanFam 3.1 assembly.
(DOCX)

S2 Table Control dogs in whole genome sequencing analysis. (XLSX)

S3 Table Summary of primer sequences used for the variant validation. (XLSX)
