# Supplementary material for: ANLN truncation causes a familial fatal acute respiratory distress syndrome in Dalmatian dogs
Source: PLoS Genet. 2017 Feb 21;13(2):e1006625. doi: 10.1371/journal.pgen.1006625 (PMC5340406; doi:10.1371/journal.pgen.1006625)
Supplement: S1 Table — The positions refer to the CanFam 3.1 assembly. (DOCX) [file pgen.1006625.s001.docx]

| **Chr** | **Marker 1** | **Marker 2** | **Start** | **Stop** | **Size (kb)** |
| --- | --- | --- | --- | --- | --- |
| 1 | BICF2P6706 | BICF2S23727073 | 117480794 | 121221371 | 3740.58 |
| 1 | BICF2G630790082 | BICF2P622123 | 94257516 | 95543662 | 1286.15 |
| 2 | BICF2P845616 | BICF2S23529489 | 80350547 | 84805989 | 4455.44 |
| 2 | BICF2P335094 | TIGRP2P30705_RS8958009 | 72916062 | 74306042 | 1389.98 |
| 2 | BICF2P11521 | BICF2P1147408 | 56460498 | 64205575 | 7745.08 |
| 4 | BICF2P1336571 | BICF2S23352557 | 57607112 | 57867028 | 259.916 |
| 5 | BICF2S23313764 | BICF2P663601 | 1305716 | 2444593 | 1138.88 |
| 6 | BICF2S23911581 | BICF2P1239210 | 24348663 | 26256433 | 1907.77 |
| 7 | BICF2G63083843 | BICF2G63084627 | 66057549 | 67203789 | 1146.24 |
| 8 | BICF2S23642757 | BICF2S23312684 | 63840372 | 70167045 | 6326.67 |
| 10 | BICF2S23418007 | BICF2S2441307 | 20120177 | 21884297 | 1764.12 |
| 10 | BICF2P790614 | BICF2P504911 | 6390240 | 8965449 | 2575.21 |
| 11 | BICF2P497190 | BICF2P890187 | 62825207 | 64839159 | 2013.95 |
| 11 | BICF2P566079 | BICF2S23048374 | 43061474 | 44766831 | 1705.36 |
| 11 | BICF2P809203 | BICF2S23525492 | 34978821 | 39320039 | 4341.22 |
| 11 | BICF2S23410619 | BICF2P867682 | 27541310 | 29545718 | 2004.41 |
| 12 | BICF2P471126 | BICF2P1234587 | 40746099 | 42854831 | 2108.73 |
| 14 | BICF2P252510 | BICF2G630531380 | 46370456 | 60959782 | 14589.3 |
| 14 | TIGRP2P187877_RS8916155 | TIGRP2P187915_RS8492724 | 16347022 | 16419392 | 72.37 |
| 15 | BICF2P914565 | BICF2G630428719 | 44034723 | 46557320 | 2522.6 |
| 15 | BICF2P892396 | BICF2P237959 | 22602656 | 24547869 | 1945.21 |
| 15 | BICF2G630439753 | BICF2G630438643 | 18863035 | 21238620 | 2375.59 |
| 16 | BICF2G630113842 | BICF2P719519 | 31929047 | 33591574 | 1662.53 |
| 17 | BICF2P855753 | BICF2G630204930 | 42781553 | 45687034 | 2905.48 |
| 18 | BICF2P1138445 | BICF2S23151349 | 54579067 | 55763074 | 1184.01 |
| 18 | BICF2P1072979 | BICF2G630689920 | 37513921 | 54156114 | 16642.2 |
| 20 | TIGRP2P273737_RS9157161 | BICF2P454193 | 25516659 | 29299066 | 3782.41 |
| 21 | BICF2S23511947 | BICF2P255736 | 24271458 | 25000611 | 729.153 |
| 21 | BICF2P294846 | BICF2G630658520 | 98812 | 6773272 | 6674.46 |
| 23 | BICF2P1287247 | BICF2G630372115 | 33175632 | 35453347 | 2277.72 |
| 24 | BICF2P331540 | BICF2S2293199 | 32674207 | 33818043 | 1143.84 |
| 24 | BICF2G630504607 | BICF2P474145 | 18170302 | 30545241 | 12374.9 |
| 24 | BICF2P1269466 | BICF2G630505716 | 3035370 | 16500565 | 13465.2 |
| 25 | TIGRP2P330476_RS9073486 | BICF2G630105132 | 38153306 | 43476369 | 5323.06 |
| 25 | TIGRP2P325209_RS8822490 | BICF2S23437181 | 13703353 | 20019610 | 6316.26 |
| 26 | BICF2G630192496 | BICF2G630191173 | 38331328 | 38939728 | 608.4 |
| 26 | BICF2P281364 | BICF2P680314 | 14157064 | 14173207 | 16.143 |
| 28 | BICF2S23738201 | BICF2P460451 | 12776332 | 14340747 | 1564.41 |
| 29 | BICF2S2292947 | BICF2S2393144 | 21939753 | 24650434 | 2710.68 |
| 31 | BICF2G630736697 | TIGRP2P376246_RS9142405 | 20012813 | 23525539 | 3512.73 |
| 31 | BICF2G630731719 | BICF2S2319185 | 9370657 | 14267478 | 4896.82 |
| 32 | BICF2G630600828 | BICF2S22960285 | 4583640 | 6063809 | 1480.17 |
| 33 | BICF2P1103100 | BICF2G63081363 | 16475338 | 17090604 | 615.266 |
| 34 | BICF2S23343185 | TIGRP2P402796_RS8869973 | 127067 | 3140892 | 3013.82 |
| 35 | BICF2P1152622 | BICF2S22935990 | 17229054 | 21034750 | 3805.7 |
| 35 | BICF2S23517309 | BICF2G630775054 | 14396375 | 15956664 | 1560.29 |
| 35 | BICF2P1293378 | BICF2G630774069 | 80309 | 10817242 | 10736.9 |
| 36 | BICF2G630765459 | TIGRP2P417008_RS9069692 | 4077615 | 6297465 | 2219.85 |
| 38 | BICF2P712659 | BICF2P1346958 | 54804 | 2832956 | 2778.15 |
